# Variation of microRNA expression in the human placenta driven by population identity and sex of the newborn

**DOI:** 10.1186/s12864-021-07542-0

**Published:** 2021-04-20

**Authors:** Song Guo, Shuyun Huang, Xi Jiang, Haiyang Hu, Dingding Han, Carlos S. Moreno, Genevieve L. Fairbrother, David A. Hughes, Mark Stoneking, Philipp Khaitovich

**Affiliations:** 1 Skolkovo Institute of Science and Technology, 121205 Moscow, Russia; 2 CAS Key Laboratory of Computational Biology, CAS-MPG Partner Institute for Computational Biology, Shanghai Institute of Nutrition and Health, CAS, 320 Yue Yang Road, Shanghai, 200031 China; 3 Department of Pathology and Laboratory Medicine and Department of Biomedical Informatics, Emory University, Atlanta, GA 30322 USA; 4 Obstetrics and Gynecology of Atlanta, 1100 Johnson Ferry Rd NE Suite 800, Center 2, Atlanta, GA 30342 USA; 5 MRC Integrative Epidemiology Unit at University of Bristol, Bristol, BS8 2BN UK; 6 Population Health Sciences, Bristol Medical School, University of Bristol, Bristol, BS8 2BN UK; 7 Max Planck Institute for Evolutionary Anthropology, 04103 Leipzig, Germany

**Keywords:** Human, Placenta, Populations, Sexual dimorphism, Newborn, Imprinting, miRNA

## Abstract

**Background:**

Analysis of lymphocyte cell lines revealed substantial differences in the expression of mRNA and microRNA (miRNA) among human populations. The extent of such population-associated differences in actual human tissues remains largely unexplored. The placenta is one of the few solid human tissues that can be collected in substantial numbers in a controlled manner, enabling quantitative analysis of transient biomolecules such as RNA transcripts. Here, we analyzed microRNA (miRNA) expression in human placental samples derived from 36 individuals representing four genetically distinct human populations: African Americans, European Americans, South Asians, and East Asians. All samples were collected at the same hospital following a unified protocol, thus minimizing potential biases that might influence the results.

**Results:**

Sequence analysis of the miRNA fraction yielded 938 annotated and 70 novel miRNA transcripts expressed in the placenta. Of them, 82 (9%) of annotated and 11 (16%) of novel miRNAs displayed quantitative expression differences among populations, generally reflecting reported genetic and mRNA-expression-based distances. Several co-expressed miRNA clusters stood out from the rest of the population-associated differences in terms of miRNA evolutionary age, tissue-specificity, and disease-association characteristics. Among three non-environmental influenced demographic parameters, the second largest contributor to miRNA expression variation after population was the sex of the newborn, with 32 miRNAs (3% of detected) exhibiting significant expression differences depending on whether the newborn was male or female. Male-associated miRNAs were evolutionarily younger and correlated inversely with the expression of target mRNA involved in neuron-related functions. In contrast, both male and female-associated miRNAs appeared to mediate different types of hormonal responses. Demographic factors further affected reported imprinted expression of 66 placental miRNAs: the imprinting strength correlated with the mother’s weight, but not height.

**Conclusions:**

Our results showed that among 12 assessed demographic variables, population affiliation and fetal sex had a substantial influence on miRNA expression variation among human placental samples. The effect of newborn-sex-associated miRNA differences further led to expression inhibition of the target genes clustering in specific functional pathways. By contrast, population-driven miRNA differences might mainly represent neutral changes with minimal functional impacts.

**Supplementary Information:**

The online version contains supplementary material available at 10.1186/s12864-021-07542-0.

## Background

Phenotypic differences among humans can be attributed to the combined effect of genetic, epigenetic, and environmental factors. The genetic basis for phenotypic variation in human populations has been extensively studied. Previous studies identified a number of genetic variants, including differences in single-nucleotide polymorphism (SNP) frequencies, copy number variation (CNV), transposable elements (TEs), and DNA methylation, that are associated with human population-specific phenotypic traits, including differential disease susceptibility [1–8].

In addition to genomic analyses, studies focusing on gene expression variation as a complex quantitative trait have played a fundamental role in advancing our understanding of the molecular mechanisms of evolution [9–11]. Most of our current knowledge about expression variation among human populations, however, comes from systematic investigations of transformed lymphoblastoid cell lines (LCLs) rather than native tissues [11–15]. Several such studies focusing on mRNA expression demonstrated that 4.5–29% of expressed genes were differentially expressed among human populations that included Europeans (CEU), Yoruba from sub-Saharan Africa (YRI), and two East Asian populations: Han Chinese (CHB) and Japanese (JPT) [12–14, 16, 17]. Parallel analysis of genetic differences explaining these expression differences identified a large number of *cis*-regulatory variants [12, 15, 16] and also *trans*-acting remote regulatory variants [14–16]. In most cases, these genetic variants might affect the binding of transcription factors (TFs) and hence alter the transcript isoform repertoire [18, 19].

MicroRNAs (miRNAs) also play a role in regulation of gene expression variation. miRNAs are short, 21–23 nucleotide-long hairpin-shaped RNA molecules that act as co-factors binding target sequences within mRNA transcripts, commonly in their 3′ untranslated regions, through Watson-Crick complementarity interactions [20–22]. Simultaneously, miRNAs interact with parts of protein complexes, functioning as RNA endonucleases or as mRNA binding proteins that sequester target mRNA from the pool of actively translated transcripts [23]. Accordingly, miRNA expression levels inversely correlate with expression levels of their mRNA targets [24, 25]. Differences in miRNA expression among human populations were examined previously using LCLs derived from CEU and YRI individuals; this study revealed population-associated expression differences for 33 of the 757 detected miRNAs, resulting in downregulation of 55–88% of their expressed target genes [26]. Cancer studies investigating circulating miRNA abundance further indicated differences between individuals of African and non-African descent [27, 28].

However, gene expression variation among human populations measured in cell lines might not be indicative of the variation found in native tissues. Earlier, we reported mRNA expression differences at 6.3% of expressed genes among placental samples, all collected at the same location following the same protocol, from four populations: African Americans, European Americans, South Asians, and East Asians [29]. Here, we build upon this work by examining microRNA (miRNA) expression in these same placental samples and how it is influenced by 12 demographic variables for which we have sufficient information. We find that population identity and sex of the newborn contribute the most to miRNA expression variation.

## Results

### Placental miRNA expression measurements

**Fig. 1:**
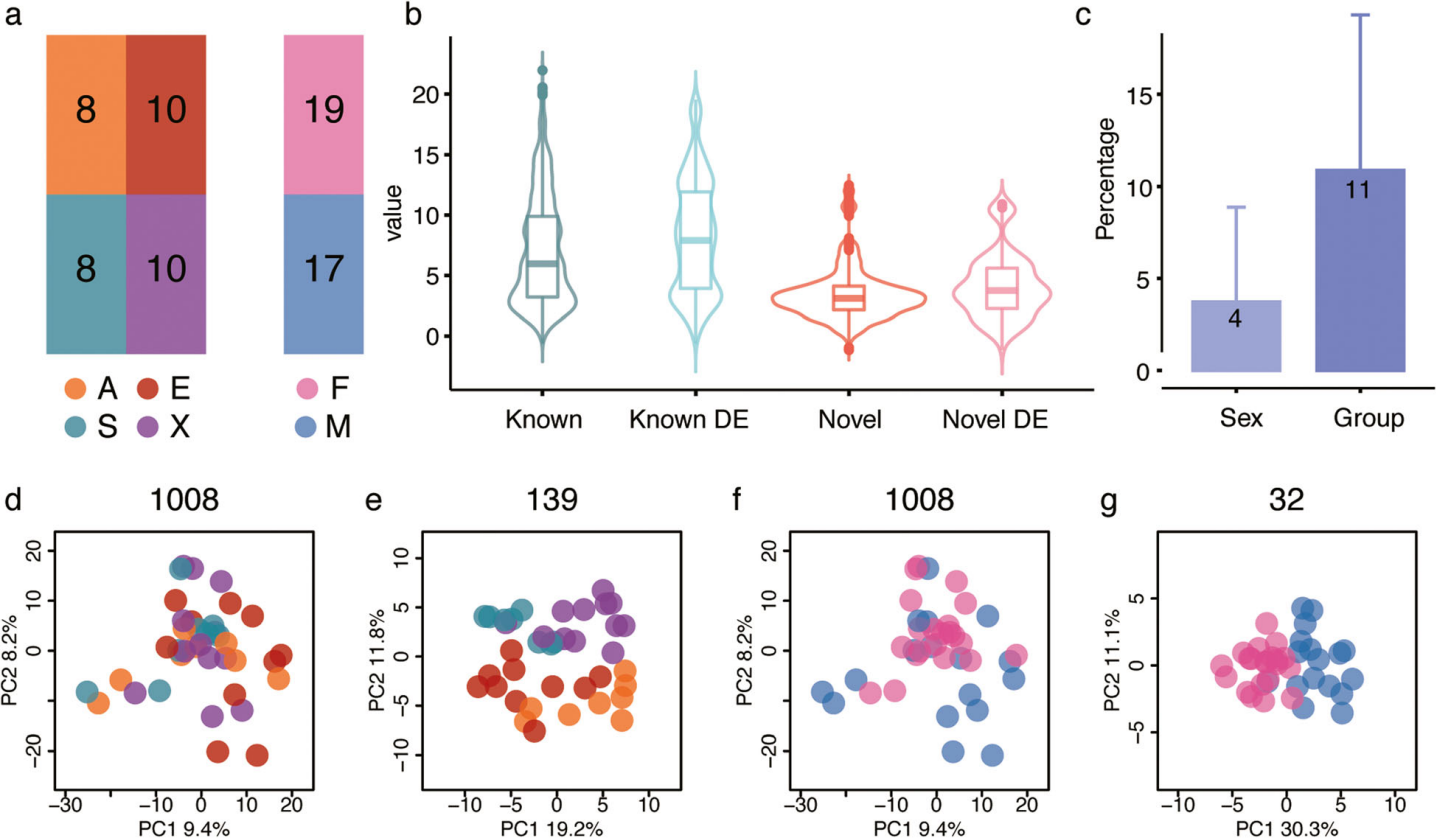
Sample information and miRNA expression distribution. **a** Schematic illustration of sample numbers according to population ID and newborn sex. The abbreviations here and in the text indicate: A – African Americans; E – European Americans; S – South Asians; X – East Asians; F - female newborn and M - male newborn. **b** Violin plot showing miRNA expression distribution. Y-axis shows the quantile normalized log2-transformed miRNA read count values after removing the batch effect. X-axis labels indicate 938 annotated miRNAs (Known), 127 annotated miRNAs differentially expressed among populations (Known DE), 70 novel miRNAs (Novel), and 12 novel miRNAs differentially expressed among populations (Novel DE). **c** Percentage of total expression variance explained by newborn sex and population. Bars represents the mean variation explained by the categorical trait. Error bars represent the standard deviation of the mean. **d-g** Principal component analysis plots based on the miRNA expression of all 1008 miRNAs (**d**, colored according to population and **f**, colored according to newborn sex), 139 miRNAs differentially expressed among populations (**e**), and 32 miRNAs differentially expressed depending on the sex of the newborn (**g**)

We analyzed miRNA expression in placenta samples from individuals representing four major human ethnic groups (further referred to as populations): African Americans, European Americans, South Asians, and East Asians (Fig. 1a). For each population, we analyzed samples from ten individuals (Additional file 1: Table S1), all from a previous study [29]. All samples were collected at the same geographic location (Northside Hospital in Atlanta, Georgia) from residents of the area. We sampled each placenta at five sites within the central *villous parenchyma* region and pooled the dissected samples before the mRNA and miRNA isolation [29]. In addition to population identity, for each sample we collected information for 26 demographic parameters from GSE66622 [29]. Among them, 12 parameters (listed in Methods), including delivery type (natural or cesarean), newborn infant’s sex, number of previous births, mother’s age, and mother’s BMI, had sufficient variability to estimate their influence on miRNA expression levels.

We estimated miRNA expression levels using high-throughput transcriptome sequencing (RNA-seq) conducted on the Illumina sequencing platform. For each sample, we obtained an average of 31.4 million reads (Additional file 2: Table S2). Based on these data, we detected 938 miRNAs annotated in miRbase (v22) and 70 novel miRNAs (Fig. 1b; Additional file 3: Table S3; Additional file 4: Table S4) with the total expression count among the 40 individuals greater than 100 reads. Four individuals did not pass data quality criteria and were removed from further analyses (Fig. 1a; Additional file 5: Fig. S1c).

### Placental miRNA expression variation

Besides individual differences, the most notable contributors to the miRNA expression variation were population identity and sex of the newborn (SON), explaining 11 and 4% of the total variation, respectively (Fig. 1c). Accordingly, 139 miRNAs showed significant expression differences among populations, including 12 novel ones (ANOVA F-test, nominal *p* < 0.05, FDR < 36%; permutation *p* < 0.0001; Fig. 1b,d,e), while 32 miRNAs, including one novel miRNA, differed depending on SON (ANOVA F-test, nominal *p* < 0.01, FDR < 31%; permutation *p* < 0.05; Fig. 1f,g). The other variables, including mother’s BMI, gestational length, gestational weight, and mother’s age did not have a significant effect on miRNA expression (Linear regression model on each variable, nominal p < 0.05, FDR > 50%, permutation *p* > 0.05).

### Population-associated placental miRNA

**Fig. 2:**
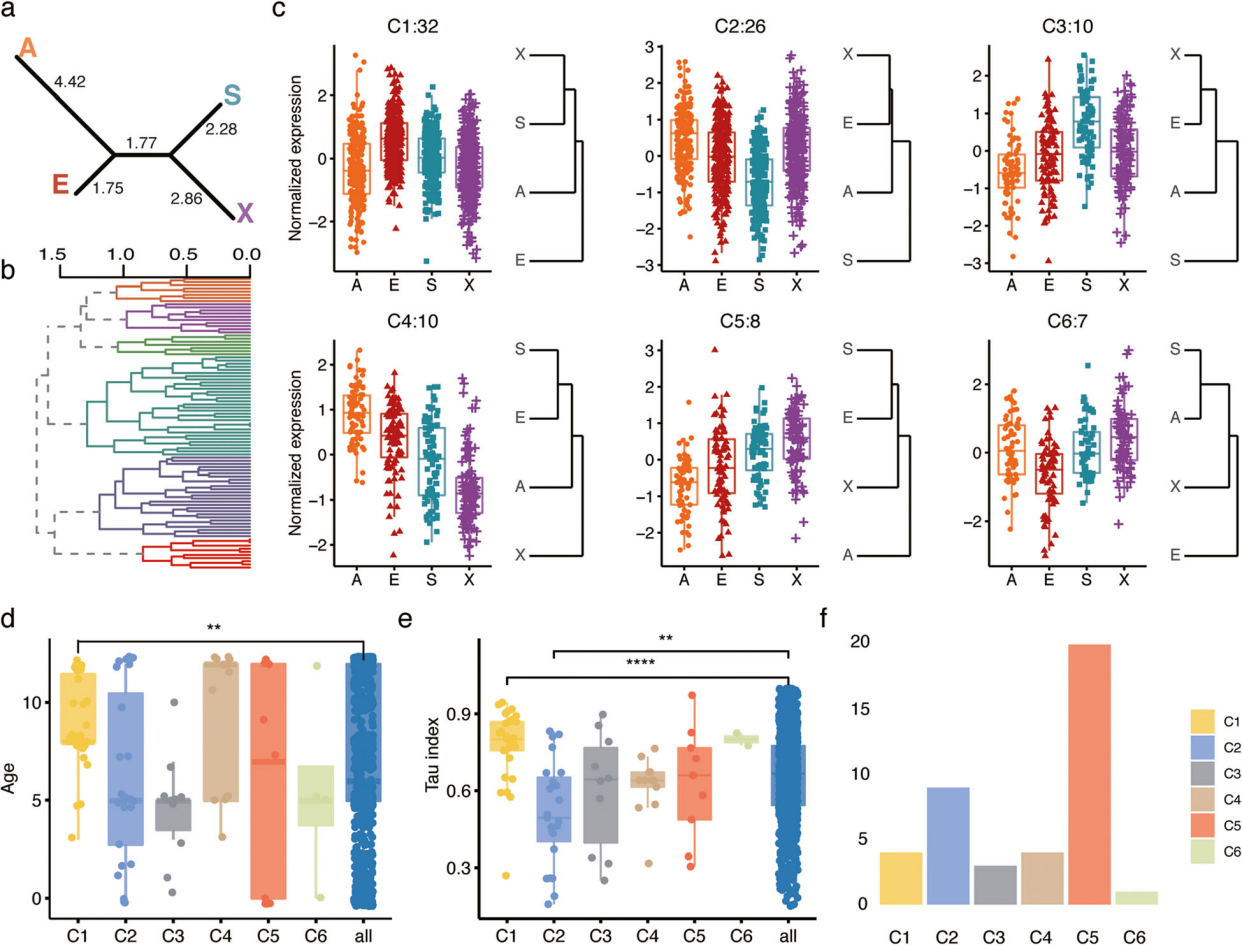
Characterization of miRNAs differentially expressed among human populations. **a** Dendrogram based on expression levels of 93 population-associated miRNAs. The abbreviations here and in the text indicate: A – African Americans; E – European Americans; S – South Asians; X – East Asians. Numbers indicate the branch length. **b** Hierarchical clustering of 93 population-associated miRNAs based on correlation of their expression profiles. Colors represent six main clusters. **c** miRNA expression patterns in each of the six clusters. Colors represent populations. Panel titles show the cluster name and the number of miRNAs in the cluster. Y-axis indicates Z-transformed miRNA expression values. The dendrograms on the right of each panel represents the average normalized expression distances among populations based on the expression of cluster miRNAs. **d** Distribution of miRNA evolutionary age in the six clusters. The age scale extends from 433 Mya (age 0) to human-specific miRNA (age 12). Asterisks indicate the significance of the difference (two-sided Wilcoxon test, ** represents nominal *p* < 0.01). **e** Distribution of miRNA tissue expression index (Tau) in the six clusters. Large values represent greater expression tissue-specificity. Asterisks indicate the significance of the difference (two-sided Wilcoxon test, **** represents nominal *p* < 0.0001). **f** Number of miRNAs associated with disease in each cluster

Further analysis of the 139 miRNAs showing population-associated expression yielded 93 miRNAs with significant expression differences between at least one pair of populations (Student’s t-test, Benjamini-Hochberg corrected p < 0.05). Visualization of the distances among populations based on the expression of 93 or 139 population-associated miRNAs yielded dendrograms compatible with the genetic relationships among populations (Fig. 2a; Additional file 6: Fig. S2 and Additional file 7: Fig. S3). Specifically, miRNA expression in African Americans was the most distant from the other populations, while the two Asian populations were most similar to one another. Similarly, miRNA expression in African American population differed most from the other three based on analysis of 1008 expressed miRNAs (Additional file 6: Fig. S2a).

Using unsupervised analysis of the 93 population-associated miRNA we identified six co-expressed miRNA clusters (Fig. 2b,c; Additional file 8: Table S5). Characterization of these clusters concerning miRNA evolutionary age, expression tissue-specificity, and disease associations further identified specific miRNA clusters showing significant feature enrichment (two-sided Wilcoxon test, nominal *p* < 0.01). Specifically, cluster 1 (C1), characterized by elevated expression in European American samples, contained significantly younger miRNAs than the bulk (two-sided Wilcoxon test, nominal p < 0.01) (Fig. 2c,d) and showed the highest miRNA expression tissue-specificity, restricted mainly to the placenta (Fig. 2c,e). Further, cluster 5 (C5), characterized by low expression in African Americans and elevated expression in Asian populations (Fig. 2c), showed the highest number of miRNA disease associations (Fig. 2f; Additional file 9: Fig. S4; Additional file 10: Table S6).

To assess the potential effects of population-associated miRNAs on expression of their target genes, we examined the published mRNA expression dataset derived from a partially overlapping set of placental samples [29] (GSE66622; Additional file 1: Table S1). Only cluster 1 (C1) reveal significant downregulation of predicted targets of population-associated miRNAs (one-side Wilcoxon rank test, *p* < 0.05, correlation *r* < − 0.5). The potential targets of C1 miRNAs were enriched in the functional term associated with vasculogenesis and muscle organ development (Additional file 11: Table S7).

### Sex-of-the-newborn-associated placental miRNA

**Fig. 3:**
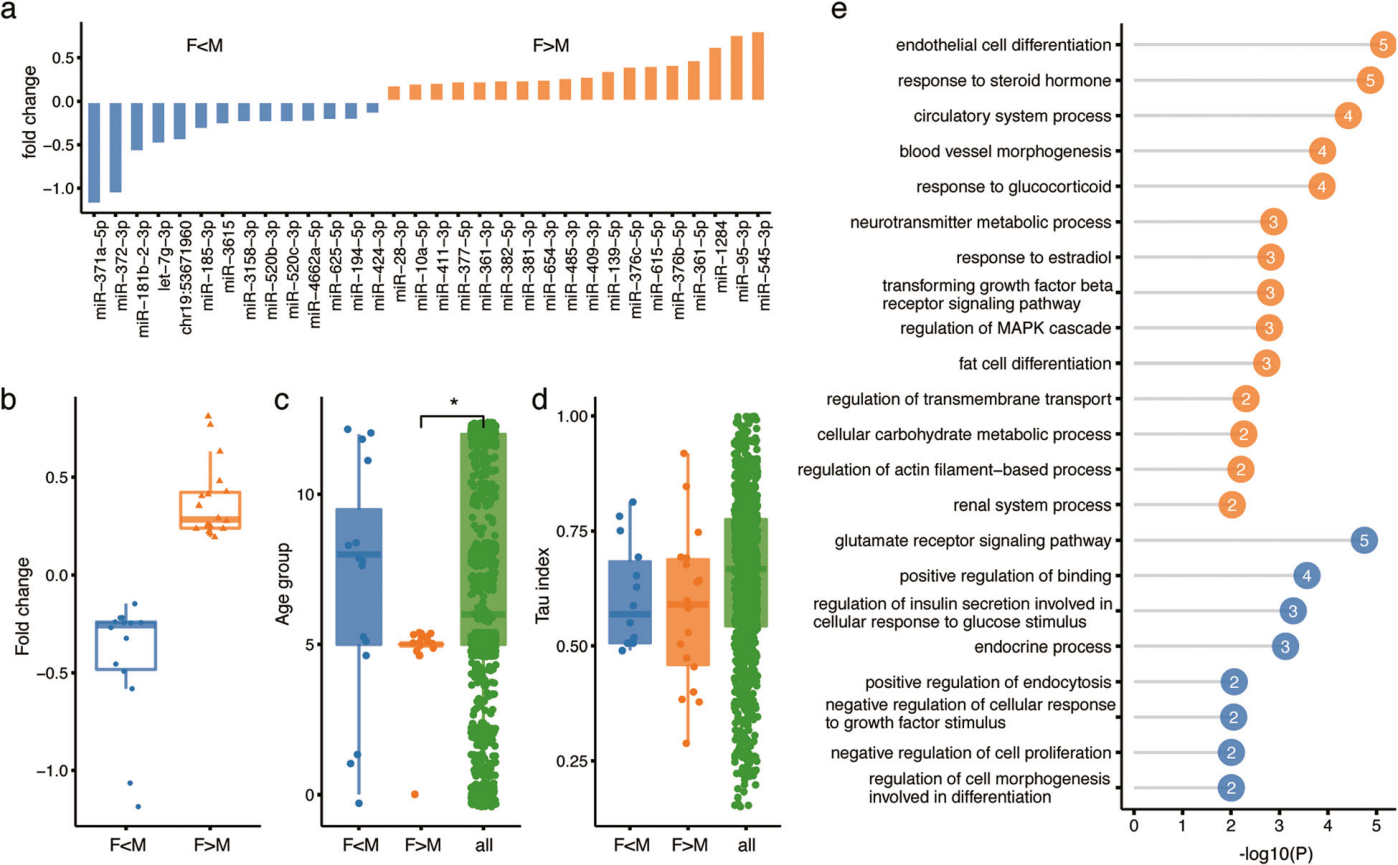
Characterization of miRNAs with newborn sex-associated expression. **a** Bar plot showing individual miRNA expression differences between placental samples from female vs. male newborn. Colors represent male-newborn-associated (F < M, blue) and female-newborn-associated (F > M, orange) miRNAs. Abbreviations: F – female newborn; M – male newborn**. b** Boxplot showing the distributions of miRNA expression fold-change for placental samples from female vs. male newborn infants. The blue and yellow boxes represent miRNAs with male-newborn-associated and female-newborn-associated expression. Each dot represents one miRNA. **c** Distribution of miRNA evolutionary age for male-newborn-associated (blue) and female-newborn-associated (orange) miRNA. The age scale extends from 433 Mya (age 0) to human-specific miRNA (age 12). Asterisks indicate the significance of the difference (two-sided Wilcoxon test, * represents nominal *p* < 0.05). **d** Distribution of miRNA tissue expression index (Tau) for male-newborn-associated (blue) and female-newborn-associated (orange) miRNA. Large values represent greater expression tissue-specificity. **e** GO terms enriched in targets of male-newborn-associated (blue) and female-newborn-associated (orange) miRNAs. X-axis and the number within circles indicate -log10-transformed *p*-values

Among 32 miRNAs showing expression differences depending on the sex of the newborn (SON-associated miRNA), 14 miRNAs were elevated in pregnancies with a male child (male-associated miRNA) and 18 in pregnancies with a female child (female-associated miRNA) (FDR < 31%, permutation p < 0.05; Fig. 3a,b; Additional file 8: Table S5). All SON-associated miRNA expression differences were reproduced in multiple populations, with 24 of the 32 reproduced in all four (Exact binomial test, *p* < 0.01; Additional file 12: Fig. S5). Notably, female-associated miRNAs were of significantly older evolutionary origin compared to most male-associated miRNAs (two-sided Wilcoxon test, nominal p < 0.05; Fig. 3c). Further, female-associated miRNAs were enriched in imprinted mir-379 cluster (C14MC) implicated in regulation of brain-specific functions [30] (hypergeometric test, Bonferroni corrected *p* = 4.58 × 10^− 6^; Additional file 10: Table S6). Both female- and male-associated miRNA groups showed, however, the same moderate tissue-specificity (Fig. 3d).

To assess the potential effects of SON-associated miRNA expression, we identified their potential targets in the published mRNA expression dataset derived from a partially overlapping set of placental samples [29] (Additional file 1: Table S1; GSE66622). In total, we classified 46 mRNAs as potential targets of male-associated miRNAs and 65 mRNAs as potential targets for female-associated miRNAs, using a combination of miRNA target predictions and the inverse relationship of miRNA and target expression profiles as selection criteria. Notably, the potential targets of male-associated miRNAs were enriched in functional terms associated with glutamate receptor signaling and endocrine processes (Fig. 3e; Additional file 11: Table S7). By contrast, the potential targets of female-associated miRNAs were enriched in functions linked to steroid hormones, estradiol, and glucocorticoid response, as well as cell differentiation and metabolic processes (Fig. 3e; Additional file 11: Table S7).

### Expression of imprinted miRNA

**Fig. 4:**
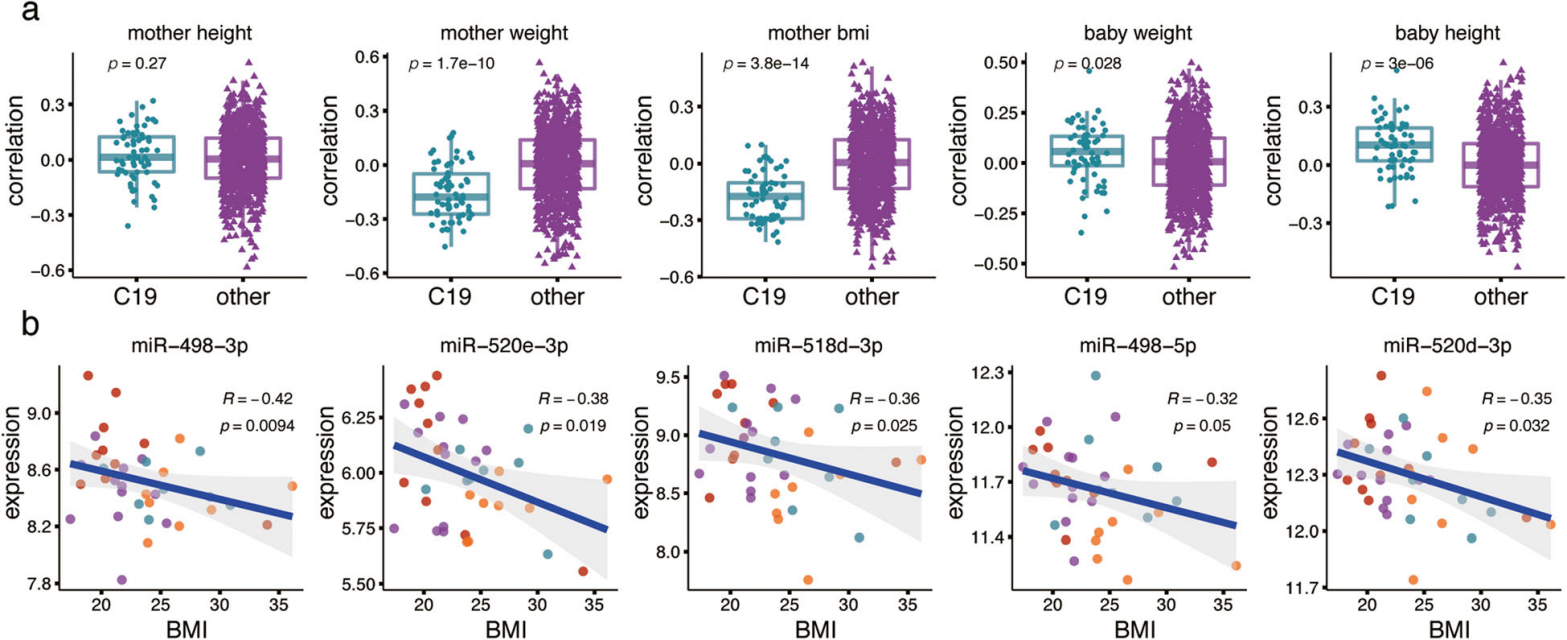
Expression of imprinted miRNAs in the C19MC cluster. **a** Correlation distribution between imprinted miRNAs located in the C19MC cluster and demographic variables. Panel titles indicate the demographic variable used in the comparison. *P*-values for a two-sided Wilcoxon test are shown within panels. **b** Five miRNAs showing a significant expression correlation with mother’s BMI. Each dot represents the expression level in a sample. Colors represent human populations as illustrated in Fig. 1a. Shaded areas represent confidence intervals. Spearman’s correlation coefficients rho (R) and p-values (p) are displayed in the top right corner of each scatter plot

One of the characteristic features of placental miRNA is the prevalence of imprinted expression, a term referring to complete or partial suppression of one of the parental alleles [31]. To assess the extent of miRNA expression imprinting in our data, we focused on the largest characterized imprinted miRNA cluster, located on chromosome 19 (C19MC) and expressed almost exclusively in the placenta [31, 32]. This cluster locus contains 67 mature miRNAs (hg38 chr19:53,665,746-53,761,746), of which 66 were detected in our study (Additional file 8: Table S5). Expression analysis of these 66 miRNAs revealed a significant negative correlation with the mother’s BMI (two-sided Wilcoxon test, *p* = 2.8 × 10^− 14^) and weight (*p* = 1.7 × 10^− 10^), but not height (*p* = 0.27) (Fig. 4a). This relationship was further apparent at the level of individual miRNAs (Spearman correlation, *p* < 0.05; Fig. 4b).

## Discussion

The placenta plays an essential role in fetal development. Thus, understanding the role of factors determining miRNA expression variation in this tissue can shed light on the fundamental mechanisms of human developmental regulation and variability. Our study design helps to address this question by minimizing sampling effects on the results. The placentas were obtained from a single location, all processed according to the same protocol, and all collected at the same time point (birth). Sampling was further averaged in each individual by taking five independently dissected tissue fragments. For each sample, we recorded 26 demographic variables relating to mothers and newborn infants, allowing us to assess their influence on placental miRNA expression variation.

Our results demonstrate that of three investigated non-environmental demographic variables, two substantially influence the expression of common posttranscriptional regulators, miRNAs, in the human placenta: population identity and sex of the newborn. Population has the most substantial influence explaining up to 11% of the total miRNA variance, and the relative miRNA expression divergence among four populations investigated in the study is consistent with their genetic divergence (Two-sided Mantel permutation test, Spearman’s correlation coefficients rho = 0.771, *p* = 0.08; Additional file 7: Fig. S3) [4]. Since genetic divergence is largely thought to reflect the accumulation of phenotypically neutral mutations [33], it is therefore conceivable that miRNA variation among populations is similarly influenced by the phenotypically neutral changes. This notion aligns with previous work suggesting that mRNA expression divergence includes a substantial proportion of functionally and phenotypically neutral changes [29, 34]. However, environmental/social differences between the groups sampled for this study could also contribute to the observed effect of population identity on miRNA expression. Moreover, our current study only collected as diverse a sample with respect to ancestry as feasible given sampling constraints, to determine whether human population identity would at all affect placental microRNA expression. It would be desirable to include Hispanic American and other ancestries in future studies.

Regulatory effects of population-associated miRNA expression differences estimated using mRNA expression data derived mainly from the same tissue revealed significant excess of expressional repression among predicted targets for only one of the six miRNA clusters. This result appears to contrast the reported widespread population-specific downregulation of miRNA targets described in cell lines [26]. While part of this discrepancy might be due to the limited statistical power of our study, the rest could be caused by unequal extent of the evolutionarily constraint in tissues and cell lines. As other regulators controlling multiple targets, miRNAs are under substantial evolutionary constraint [35, 36]. Assuming that most of the randomly arising population-specific miRNA expression differences are non-adaptive, those with large regulatory effects are likely to be detrimental and will not be observed in a natural tissue, such as placenta. The artificial growth conditions of the cell lines could, however, allow the manifestation of large-scale population-associated regulatory effects of miRNA variation.

Several technical factors might have further restricted our ability to detect miRNA-driven regulation of their predicted mRNA targets. Such factors include a mismatch between computational and experimentally verified miRNA target predictions, sequestering of target mRNA out of the translational pool without degradation, and the complex and often a tissue-specific interplay between miRNAs and other regulators [37, 38]. Biologically, our study includes a limited number of populations and biological replicates and certainly does not cover all population-associated aspects of miRNA regulatory effects. Evolutionarily, as mentioned above, the proportion of population-associated miRNA differences leading to functionally meaningful effects might be minor, analogous to genetic and mRNA divergence [4, 29]. It has to be noted, however, that despite these limitations, the fact that our study reveals many population-associated miRNA expression differences indicates the importance of further studies investigating the functional significance of this phenomenon.

Previous investigation of mRNA expression in human placenta reported 41 genes with sex-associated expression, 12 of them (30%) localized on sex chromosomes [39]. The substantial prevalence for sex chromosome localization was not, however, the case for SON-associated differences in miRNA expression: of 32 miRNAs, four (13%) localize on sex chromosomes. Sex-associated differences in miRNA expression were similarly reported in human tissues other than the placenta. Specifically, miRNA analysis across postnatal brain development revealed 40 miRNAs with significant sex-biased expression differences in the prefrontal cortex regions, 93% of them female-biased [40]. Further, investigation of four adult human tissues – brain, colorectal mucosa, peripheral blood, and cord blood – revealing 73 female-biased and 163 male-biased expressed miRNAs [41]. Notably, two of 32 SON-associated miRNAs overlapped with miRNAs showing corresponding sex-biased expression in the adult brain, and two overlapped with miRNAs showing such a bias in the peripheral blood. In addition to human studies, sex-biased miRNA expression was reported in mouse brain [42], mouse liver [43], rat liver [44], developing rat cortex [45], and other mammalian somatic tissues [46]. Previous studies singled out hormonal regulation as the main driving mechanism of miRNA sex-biased expression [43, 47]. In our study, functional analysis of target genes downregulated by SON-associated miRNAs in placenta similarly revealed terms related to hormonal processes, but also in other biological pathways.

In addition to the identification of population and SON effects, our data allowed us to examine a well-characterized phenomenon of imprinted miRNA expression in the human placenta [31]. Previously reported imprinted expression of the miRNA cluster located on chromosome 19 (C19MC) [31, 32] was also evident in our data. Previous work further linked the amplitude of the imprinting effect with the mother’s BMI [48]. Our analysis of demographic variables indicated that the relationship between C19MC cluster imprinting and mother’s BMI depends on the mother’s weight but not the height.

## Conclusions

Our results indicate that miRNA expression in the placenta varies substantially due to the population identity and the sex of the newborn. While the majority of population effects might reflect recent evolutionary drift caused by geographical separation, miRNA expression differences associated with female newborns are evolutionarily older than those associated with male newborns and display detectable regulatory effect on genes involved in particular functional processes. Further, the identification of regulatory effects induced by population-associated miRNA expression differences might differ between tissues and cell lines, highlighting the need for further studies of miRNA variation in a population framework.

## Methods

### Samples

Placental samples were obtained from a previously study [29], and included ten individuals from each ancestry (African American, European American, South Asian American (India) and East Asian American ancestry (Korea, China, Vietnam, and Taiwan)) from Northside Hospital in Atlanta, Georgia with the approval of the Northside Hospital Institutional Review Board (NSH #804), as described previously [29]. The placenta dissection procedure was described in detail in the previous study [29]. In short, we dissected centrally located villus parenchyma tissue, avoiding the decidua, chorion, and amnion, at five non-adjacent locations of each placenta and pooled them into a single sample to homogenize the cell types composition among samples. The sample information with 26 demographic variables was provided under GEO (GSE66622).

### Construction of indexed small RNA-Seq libraries

Total RNA was isolated from human placenta using Trizol (Invitrogen, USA) according to the manufacturer’s directions. RNA quality was determined using Agilent 6000 Nano chips on an Agilent Technologies 2100 Bioanalyzer. Samples with RIN > 6 were selected for library construction. Sequencing libraries were constructed in a single batch according to the TruSeq SmallRNA Libary Preparation guide (Illumina) with no modification. In each sequencing lane on an Illumina HiSeq X10 platform, we pooled 14 samples with different index sequences and carried out 150-bp paired-ended sequences. We randomly distributed 10 African American, 10 European American, 10 South Asian American, and nine East Asian American samples into three sequencing lanes. We further included the tenth East Asian American sample (X14) into each of the three lanes to control for potential sequencing artifacts among the lanes (Additional file 1: Table S1).

### Sequence mapping and novel miRNA identification

The adapter sequence at the 3′-end of each read was trimmed using cutadapt v1.13 [49] with parameters -m 17 -M 50 -a TGGAATTCTCGGGTGCCAAGG -A GATCGTCGGACTGTAGAACTCTGAAC. Novel miRNAs for each sample were detected using the miRDeep2 algorithm with default parameters based on the human genome (hg38) and miRbase v22 as references [50]. Predicted rRNA/tRNA reads and reads with the miRDeep2 score < =5, representing the miRNA hairpin properties matching, were removed from the following analyses. Sequencing reads mapped to overlapping miRNA genomic locations were merged across all samples. The detected miRNAs with no overlap with known mature miRNA genomic positions were considered as novel.

### miRNA expression quantification

The trimmed raw sequences that were at least 17 bp long were mapped allowing no mismatches to the sequences of known and novel mature miRNAs determined as described in the previous section, extending 8 nt both up and downstream, using the Bowtie algorithm [51]. To quantify miRNA expression, only the reads from the R1 strand were considered. Following the protocol described in [52], the expression value of each miRNA was calculated as the number of reads mapping to the reference sequence for the mature miRNA. All miRNA read count data were log2 transformed and quantile normalized. As sequences were obtained in three batches, the batch effect, identified by principal component analysis, was removed by the removeBatchEffect (limma) algorithm (Additional file 5: Fig. S1). The detailed read mapping information is listed in Additional file 2: Table S2. Based on a principle component analysis of all 42 samples, four samples were considered as outliers based on their dispersion and were removed from further analysis (Additional file 5: Fig. S1c). In total, data from 8 African American, 10 European American, 8 South Asian American and 10 East Asian American individuals were retained.

### Estimation of miRNA expression variation

Of the 26 demographic variables provided in the previous study [29](GSE66622), 12 had at least five observations for each level. These 12 variables include: birth delivery type (Cesarean or natural), sex of the newborn, maternal body mass index, number of pregnancies, has pregnancy infection or not, first pregnancy or not, mother’s age, birth length of the newborn child, birth weight of the newborn child, number of children, drinker or not, and population identity. For these variables we estimated miRNA expression variation using a multivariate Type I analysis of variance with the above sequential ordering, denoted as Model1 (ANOVA; aov() function in the R stats package; Additional file 13: Table S8). The variation explained by each factor was estimated by an eta-squared statistic using the sums of squares.

In a second model (Model2), we recalculated the eta-squared statistics after subtracting the potential effects of four continuous demographic variables using linear regression. The four continuous variables modeled first were: maternal body mass index, birth weight of the newborn child, mother’s age and birth length of the child. The residuals for each miRNA were carried forward into subsequent linear regressions that included birth delivery type (Cesarean or natural), sex of the newborn and population identity. The estimations of variation explained agreed well between model1 and model 2 (Fig. 1c). Model2 as described here was used to identify miRNAs with population and sex of a child effects.

### Identification of population-associated miRNA expression differences

To identify miRNAs differentially expressed between populations, we applied an ANOVA test with factors contributing the most to the general variation effects listed in the model in the following order: birth delivery type, sex of the newborn, and population. The expression data used in this analysis were adjusted for the continuous demographic variables as described in the previous section. To estimate the significance of results, we applied the same test 1000 times to the dataset with randomly permuted individual labels. The permutation *p*-value was calculated as the proportion of times in which the number of significantly differentially-expressed miRNAs was greater than or equal to the observed difference in the data.

To further identify the miRNAs differentially expressed between any two human populations, we applied a two-sided pairwise t-test (pairwise.t.test in R multcomp package). Differentially-expressed miRNAs were defined as those with a *p* < 0.05 after Benjamini-Hochberg correction.

### Clustering of population-associated placental miRNA

To detect co-expression patterns among 93 population-associated miRNAs, we applied a hierarchical clustering method (hclust function in R) on z-transformed miRNA expression values with (1-rho) as the distance measure, where rho is the Spearman correlation coefficient. To define miRNA clusters we used the cutree() function (from the R stats package) and set k, the number of clusters, to six. The choice of k = 6 was based on visual inspection of the dendrogram (Fig. 2b).

### Construction of population dendrograms

To illustrate the extent of the expression difference among human populations, we built unrooted neighbor joining trees (nj function in R package ape) using the Euclidean distances between the mean expression of each miRNA in each population. The expression level of each miRNA was scaled by the mean expression of this miRNA across all samples. A single population tree was generated by estimating the mean Euclidean distance across all miRNAs (Additional file 6: Fig. S2a). A differentially expressed (DE) tree was generated by taking the mean Euclidean distance across 93 DE miRNAs (Fig. 2a) or 139 DE miRNAs (Additional file 6: Fig. S2b). The same approach was applied to human genetic data with distances based on the Fst values from the 1000 genome project [4]. Populations used for genetic data included African Americans, CEPH, Telugu, Han Chinese, Southern Han Chinese, and Kinh Vietnamese to match the populations used in our study. The mean Fst value of the Han Chinese, Southern Han Chinese, and Kinh Vietnamese populations was considered as the value for the East Asian population.

### Quantification of miRNA expression tissue specificity

We downloaded 87 miRNA expression datasets derived from 12 healthy tissues from miRmine database [53]. Tissue specificity was measured by the tau index [54] using quantile normalized log2-RPM expression data. A tau value can range between 0 for house-keeping genes and 1 for tissue-specific genes.

### Dating of human miRNA evolutionary ages

To estimate miRNA evolutionary age, we first downloaded all hairpin sequences for the 23 species listed in Additional file 14: Table S9 from miRBase v22. We then mapped the hairpin sequences to all of the 23 genomes using blastn with parameter -evalue 1e-5. A positive hit was called when the sequence overlapped with another species’ known miRNA coordinates using bedtools intersect with default parameters. If a miRNA pair could be blasted reciprocally, it was considered to be a one-to-one miRNA hit. Next, according to Additional file 14: Table S9, we assigned each miRNA to an age group from 0 to 12, where 0 is the oldest and 12 is the most recent evolutionary age group. We considered the evolutionary age of the pre-miRNA to correspond to the evolutionary age of the mature miRNA.

### miRNA target prediction and functional analysis of miRNA targets

We downloaded miRNA predicted targets using miRNAtap [55], requiring a predicted target to be identified by at least three of the five following methods: “pictar”, “diana”, “targetscan”, “miranda” and “mirdb”. To get potential targets for each cluster of population-associated miRNAs, we assessed if predicted target genes for a cluster of miRNAs were negatively regulated compared to non-target genes (Spearman correlation coefficient rho<− 0.5; one-sided Wilcoxon rank test *p* < 0.05). To identify potential targets of SON-associated miRNAs, we required the absolute expression fold change of miRNA targets to be greater than 0.1 and the direction of change to be opposite to the direction of miRNA expression difference. The normalized expression of miRNA targets was obtained from [29].

Gene ontology (GO) and pathway enrichment tests were processed with Metascape [56] with all genes expressed in human placenta used as a background. The GO terms with -log_10_(p) > 2 were reported as significantly enriched.

### miRNA disease association analysis

We used the database TAM2.0 [57] to analyze the functional and disease associations of miRNAs with cancer-related terms masked. All expressed human placenta miRNAs were taken as a background. The terms with nominal p < 0.05 were reported as associated diseases.

### Statistical analysis and software

All statistical analyses and plots were performed in the R environment (http://www.r-project.org/), using packages preprocessCore, multcomp, limma, dendextend, ggpubr, ggsci, ggplot2, gridExtra, reshape2, miRNAtap and cultevo. TAM2.0 was used for miRNA disease association and Metascape was used for the miRNA target enrichment test.

## Supplementary Information

**Additional file 1: Table S1**. Sample list.

**Additional file 2: Table S2**. List of miRNA sequencing files and statistics of mapping.

**Additional file 3: Table S3**. Quantified miRNAs’ expression by counts.

**Additional file 4: Table S4**. Quantile normalized miRNA expression after remove batch effect.

**Additional file 5: Figure S1**. miRNA expression variation among 40 individuals. **a** Principal component analysis plots based on the miRNA expression of all 1008 miRNAs in 40 individual placental samples before removing batch effect. Colors indicate sequencing batch. Each dot represents a sample. **b** Colors indicate human populations: orange – African Americans; red – European Americans; light blue – South Asians; purple – East Asians. Each dot represents a sample. **c**. Principal component analysis plots based on the miRNA expression of all 1008 miRNAs in 40 individual placental samples after removing batch effect. Colors indicate human populations: orange – African Americans; red – European Americans; light blue – South Asians; purple – East Asians. Each dot represents a sample; Red arrows point to four outliers removed from further analysis.

**Additional file 6: Figure S2**. Dendrograms based on miRNA expression. **a** Dendrogram based on expression of 1008 detected miRNAs. **b** Dendrogram based on expression of 139 population-associated miRNAs. The abbreviations indicate human populations: A – African Americans; E – European Americans; S – South Asians; X – East Asians. Numbers indicate the branch length.

**Additional file 7: Figure S3**. Dendrogram of the genetic divergence among four human populations**.** Shown is a neighbor joining tree based on Fst values from the 1000 genomes project. Populations used in the analysis include African American, CEPH, Telugu, Han Chinese, Southern Han Chinese, and Kinh Vietnamese, to match the populations used in our study. The mean Fst value of the Han Chinese, Southern Han Chinese, and Kinh Vietnamese populations was considered as the value for East Asian population. The abbreviations indicate human populations: A – African Americans; E – European Americans; S – South Asians; X – East Asians. Numbers indicate the branch length.

**Additional file 8: Table S5**. List of miRNAs on the cluster of C1-C6, SON, and C19MC.

**Additional file 9: Figure S4**. Enrichment for differentially-expressed miRNAs in specific disease categories. Enrichment for miRNAs differentially expressed among human populations (clusters C1–6) or depending on the sex of the newborn (F > M and F < M) among miRNAs associated with human diseases according to the TAM2.0 database with exclusion of cancer-related terms [57]. Color represents the value of -log(*p*-value). Only the cases with enrichment nominal *p* < 0.05 are highlighted. The details are listed in Additional file 10: Table S6.

**Additional file 10: Table S6**. miRNA associated disease and functions from TAM2.0.

**Additional file 11: Table S7**. Enriched functions tested on miRNA targets from Metascape.

**Additional file 12: Figure S5**. Expression of newborn-sex-associated miRNA in each human population. Each bar represents the expression difference between placental samples from female newborn vs. male newborn, displayed separately for each population. Colors represent male-newborn-associated (F < M, blue) and female-newborn-associated (F > M, orange) miRNAs. The abbreviations here and in the text indicate: A – African Americans; E – European Americans; S – South Asians; X – East Asians.

**Additional file 13: Table S8**. Variance proportion of 12 demographic factors.

**Additional file 14: Table S9**. List of species and age groups used in dating the evolutionary age of miRNAs.

## Data Availability

The raw datasets generated during the current study are available in the Sequence Read Archive (SRA, https://www.ncbi.nlm.nih.gov/Traces/study/?acc=PRJNA602793) with accession number PRJNA602793. Detailed sample information can be found both in the PRJNA602793 and in the Gene Expression Omnibus (GEO) under accession number GSE66622 (https://www.ncbi.nlm.nih.gov/geo/query/acc.cgi?acc=GSE66622). The quantified miRNA counts, and normalized expression data can be found in supplementary files (Additional file 3: Table S3; Additional file 4: Table S4). The quantified gene expression data from the previous study [29] were downloaded from GEO under accession number GSE66622 (https://www.ncbi.nlm.nih.gov/geo/query/acc.cgi?acc=GSE66622). miRNA expression datasets derived from 12 healthy tissues were downloaded from miRmine database under the link of https://guanfiles.dcmb.med.umich.edu/mirmine/miRmine.zip. The Fst values between human populations were downloaded from the 1,000 genome project Supplementary Information Table 5 [4].

## Abbreviations

miRNA: microRNA
BMI: Body mass index
SON: Sex of newborn
RPM: Reads per million
RNA-seq: RNA sequencing
ANOVA: analysis of variance.
MYA: Million years ago
C19MC: Chromosome 19 miRNA cluster
