# Supplementary figures and images for: Variation of microRNA expression in the human placenta driven by population identity and sex of the newborn
Source: BMC Genomics. 2021 Apr 20;22:286. doi: 10.1186/s12864-021-07542-0 (PMC8059241; doi:10.1186/s12864-021-07542-0)

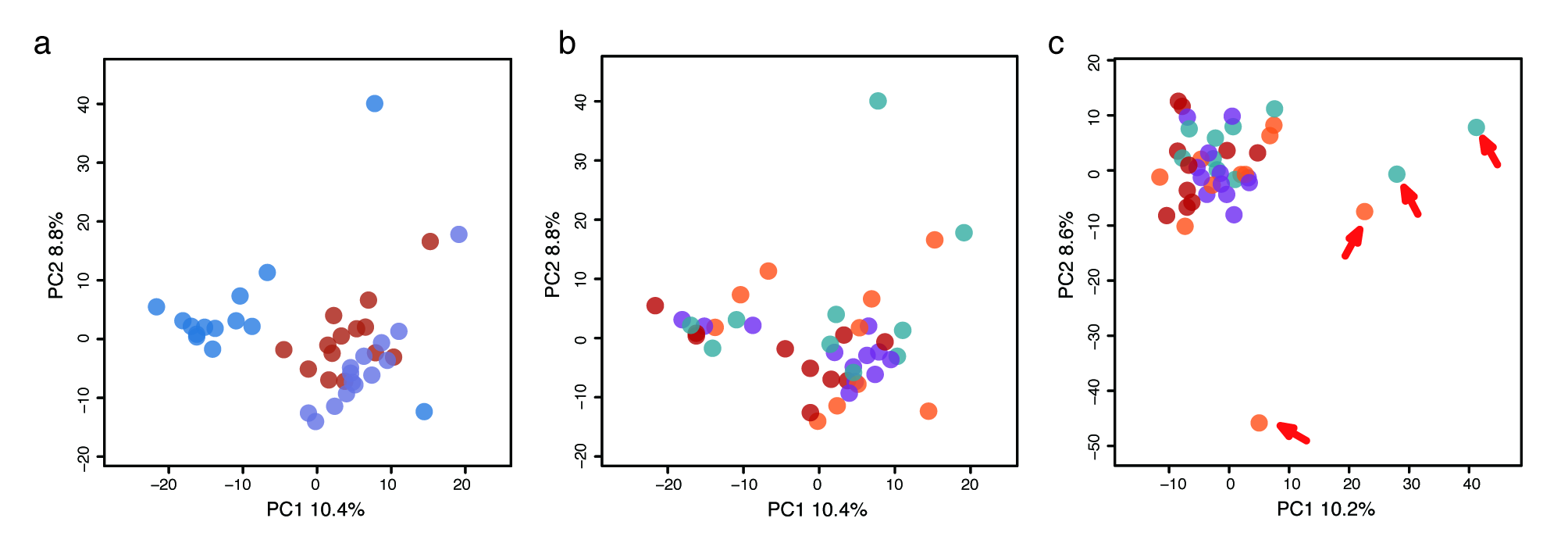

Supplement: Supplementary file 5 — Additional file 5: Figure S1. miRNA expression variation among 40 individuals. a Principal component analysis plots based on the miRNA expression of all 1008 miRNAs in 40 individual placental samples before removing batch effect. Colors indicate sequencing batch. Each dot represents a sample. b Colors indicate human populations: orange – African Americans; red – European Americans; light blue – South Asians; purple – East Asians. Each dot represents a sample. c. Principal component analysis plots based on the miRNA expression of all 1008 miRNAs in 40 individual placental samples after removing batch effect. Colors indicate human populations: orange – African Americans; red – European Americans; light blue – South Asians; purple – East Asians. Each dot represents a sample; Red arrows point to four outliers removed from further analysis. [file 12864_2021_7542_MOESM5_ESM.tif]

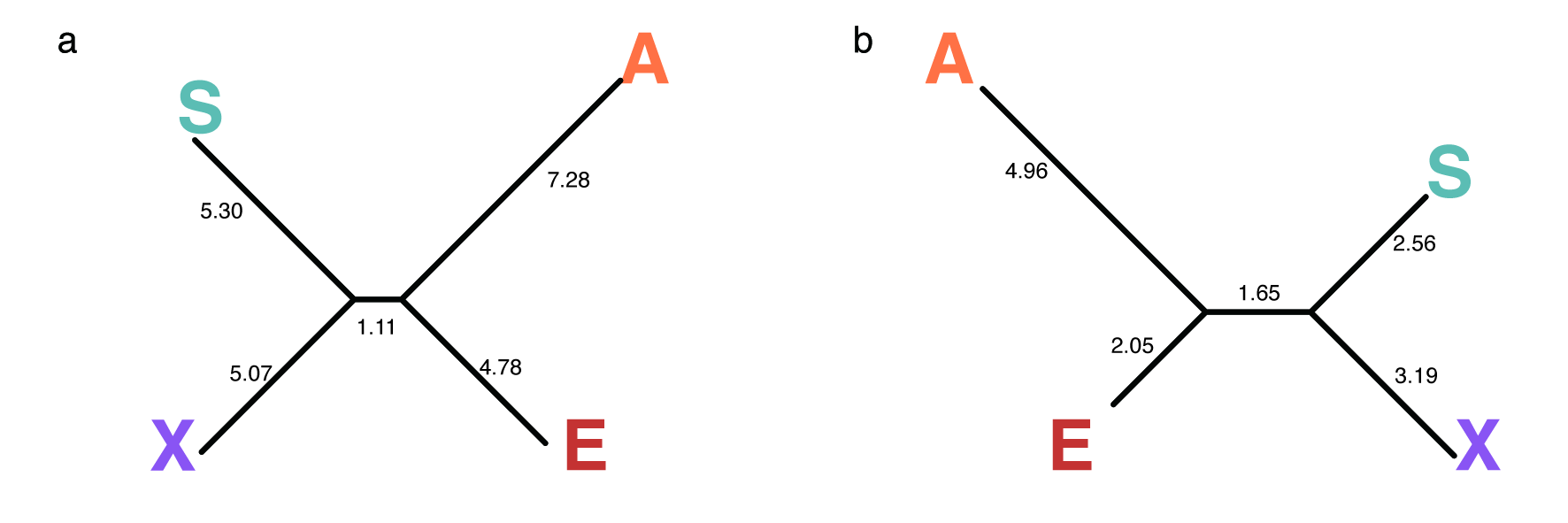

Supplement: Supplementary file 6 — Additional file 6: Figure S2. Dendrograms based on miRNA expression. a Dendrogram based on expression of 1008 detected miRNAs. b Dendrogram based on expression of 139 population-associated miRNAs. The abbreviations indicate human populations: A – African Americans; E – European Americans; S – South Asians; X – East Asians. Numbers indicate the branch length. [file 12864_2021_7542_MOESM6_ESM.tif]

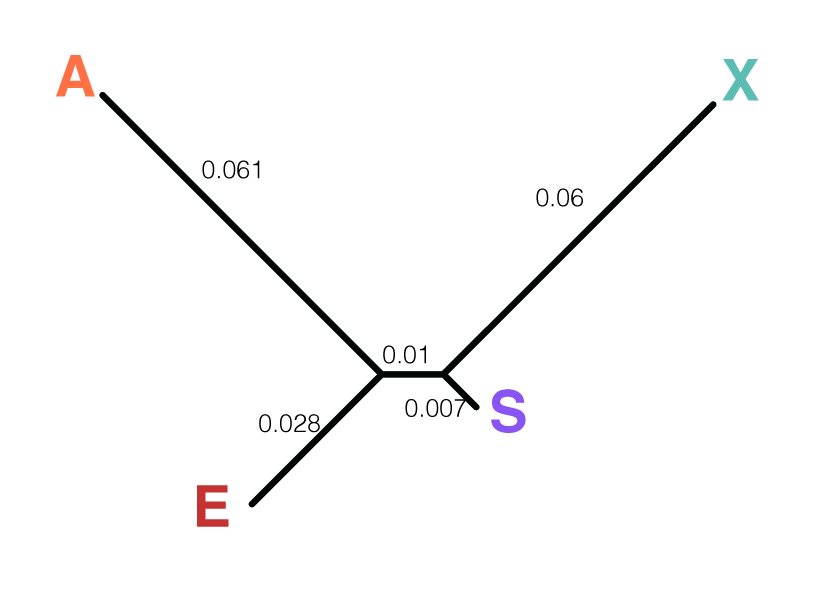

Supplement: Supplementary file 7 — Additional file 7: Figure S3. Dendrogram of the genetic divergence among four human populations. Shown is a neighbor joining tree based on Fst values from the 1000 genomes project. Populations used in the analysis include African American, CEPH, Telugu, Han Chinese, Southern Han Chinese, and Kinh Vietnamese, to match the populations used in our study. The mean Fst value of the Han Chinese, Southern Han Chinese, and Kinh Vietnamese populations was considered as the value for East Asian population. The abbreviations indicate human populations: A – African Americans; E – European Americans; S – South Asians; X – East Asians. Numbers indicate the branch length. [file 12864_2021_7542_MOESM7_ESM.tif]

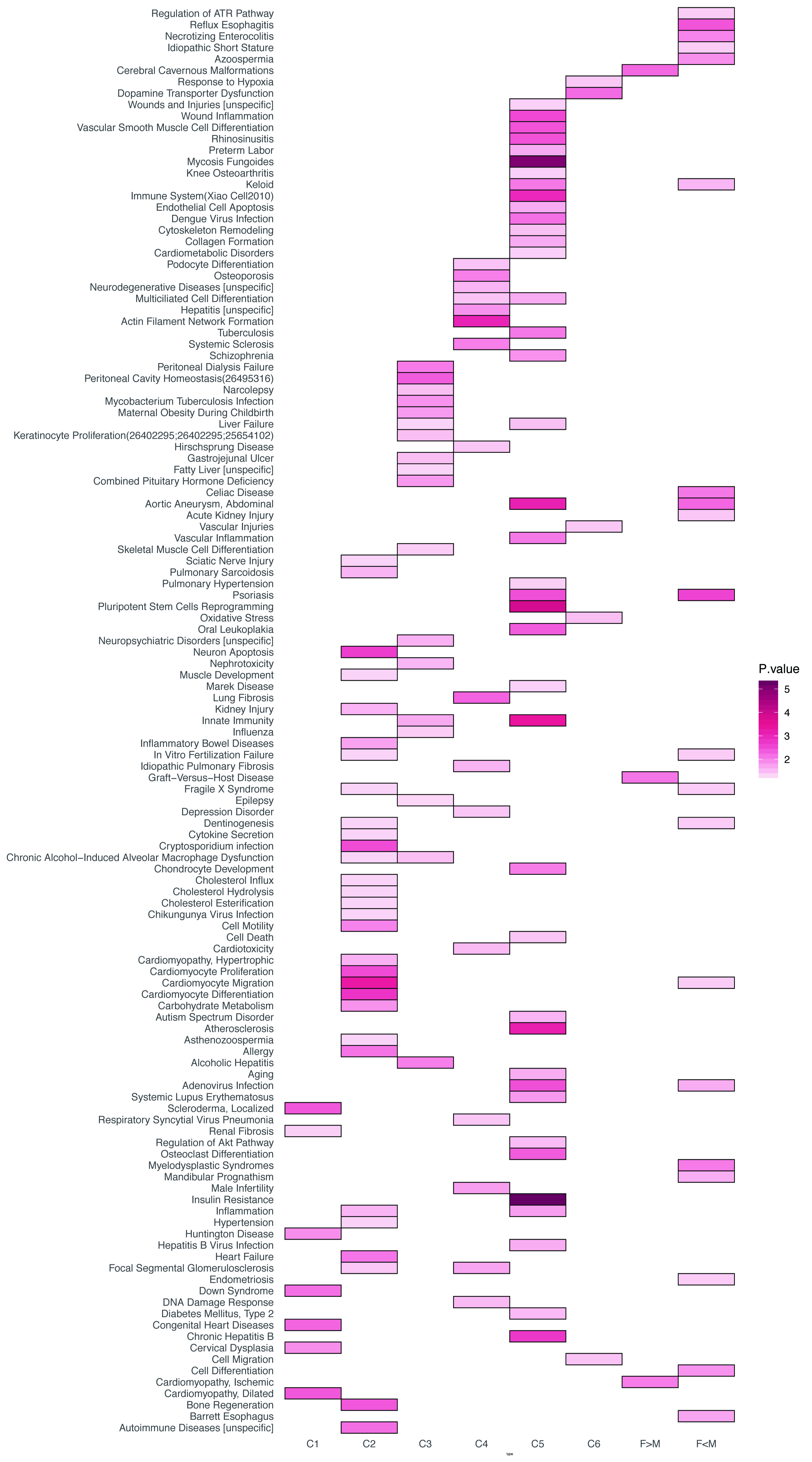

Supplement: Supplementary file 9 — Additional file 9: Figure S4. Enrichment for differentially-expressed miRNAs in specific disease categories. Enrichment for miRNAs differentially expressed among human populations (clusters C1–6) or depending on the sex of the newborn (F > M and F < M) among miRNAs associated with human diseases according to the TAM2.0 database with exclusion of cancer-related terms [57]. Color represents the value of -log(p-value). Only the cases with enrichment nominal p < 0.05 are highlighted. The details are listed in Additional file 10: Table S6. [file 12864_2021_7542_MOESM9_ESM.tif]

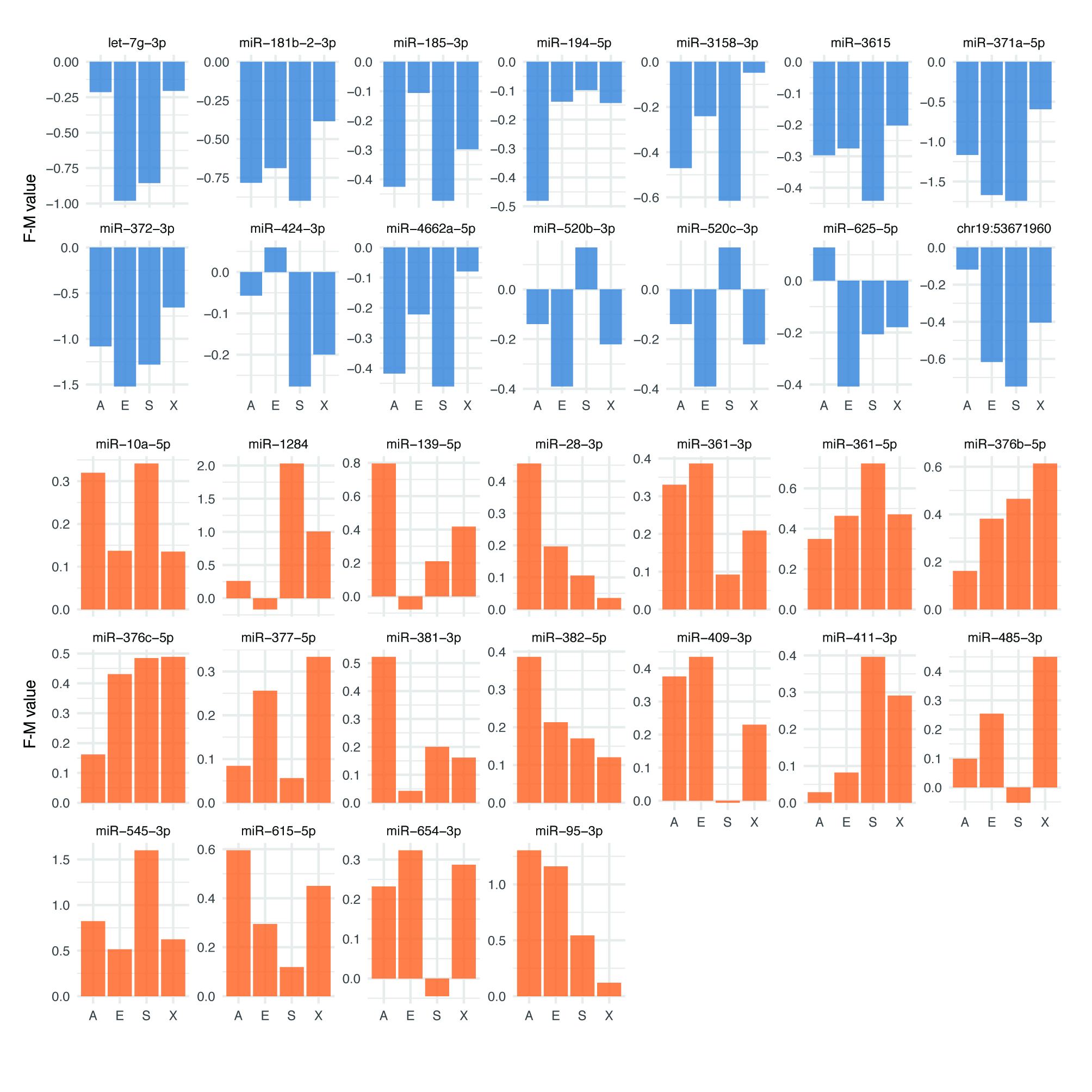

Supplement: Supplementary file 12 — Additional file 12: Figure S5. Expression of newborn-sex-associated miRNA in each human population. Each bar represents the expression difference between placental samples from female newborn vs. male newborn, displayed separately for each population. Colors represent male-newborn-associated (F < M, blue) and female-newborn-associated (F > M, orange) miRNAs. The abbreviations here and in the text indicate: A – African Americans; E – European Americans; S – South Asians; X – East Asians. [file 12864_2021_7542_MOESM12_ESM.tif]
